# Application of Electronic Nose to Discriminate Species of Mold Strains in Synthetic Brines

**DOI:** 10.3389/fmicb.2022.897178

**Published:** 2022-05-06

**Authors:** Ramiro Sánchez, Francisco Pérez-Nevado, Ismael Montero-Fernández, Jesús Lozano, Félix Meléndez, Daniel Martín-Vertedor

**Affiliations:** ^1^Technological Institute of Food and Agriculture CICYTEX-INTAEX, Junta of Extremadura, Badajoz, Spain; ^2^Área de Nutrición y Bromatología, Departamento de Producción Animal y Ciencia de los Alimentos, Escuela de Ingenierías Agrarias, Universidad de Extremadura, Badajoz, Spain; ^3^Research Institute of Agricultural Resources (INURA), Badajoz, Spain; ^4^Department of Agricultural and Forestry Engineering, School of Agrarian Engineering, University of Extremadura, Badajoz, Spain; ^5^Industrial Engineering School, University of Extremadura, Badajoz, Spain

**Keywords:** altering microorganisms, Spanish-style table olives, sensory quality, inoculation, volatile compounds

## Abstract

The chemical composition of the brine for Spanish-style table olives plays a crucial role during the fermentation process. Traditional laboratory analysis requires a high consumption of reagents, highly qualified personnel, sophisticated equipment, long analysis times, and large amounts of samples. Analysis carried out using an electronic nose (E-nose) offers an alternative, non-destructive technique and is useful in determining alterations in brines caused by microorganisms. In the present research, nine mold strains isolated from spoiled olives were inoculated in synthetic brines to determine the effect of microbial development on sensory quality, volatile profile, and the capacity of E-nose to discriminate altered brines from the healthy ones. The brines inoculated with the mold strains presented negative attributes related to aromas of mold, wood, leather, rancidity and, organic solvents among others. The highest intensity of defect was presented by the brines inoculated with the strains *Galactomyces geotricum* (G.G.2); three *Penicillium expansum* (P.E.3, P.E.4, and P.E.20); one *Penicillium glabrum* (P.G.19); three *Aspergillus flavus* (A.F.9, A.F.18, and A.F.21); and one *Fusarium solani* (F.S.11). A total of 19 volatile compounds were identified by gas chromatography. Sensory analysis allowed us to classify the synthetic brines based on the degree of alteration produced by the mold strains used. Also, the E-nose data were able to discriminate the inoculated brines regardless of the intensity of the defect. These results demonstrate the capacity of the E-nose to discriminate alterations in brines produced by molds, thereby making it a useful tool to be applied during the elaboration process to detect early alterations in table olive fermentation.

## Introduction

Spanish-style table olives are consumed in many countries. During their elaboration process, olives are first treated with alkali, and then passed through a stage of washing and fermentation in brine that gives them unique, organoleptic characteristics. However, this process of fermentation and its subsequent conservation can bring about deterioration defects associated with abnormal fermentation or environmental conditions (de Castro et al., 2022). The olive fermentation is mainly carried out by lactic acid bacteria (IOC, 2020); however, some yeasts have positive effects, producing volatile compounds and desirable metabolites that improve the sensorial characteristics (Hernández et al., 2007; Nisiotou et al., 2009; Arroyo-López et al., 2012; Hurtado et al., 2012; Zago et al., 2013; Schaide et al., 2019).

On the other hand, the fermentation process can cause the growth of filamentous fungi whenever environmental conditions are favorable, contaminating the olives and producing harmful mycotoxins such as those derived from *Aspergillus* and *Penicilium* species (Ghitakou et al., 2006). In other cases, the alteration occurs because the spores reach the brines and cause fungal growth to develop during the storage of the olives, affecting their appearance, flavor, taste, and texture (Franzetti et al., 2011). For this reason, process control precautions, such as good handling, storage temperature, salinity, and packaging processes should be recommended to avoid the development of fungi and mycotoxin throughout the entire table olive process (Bavaro et al., 2017). In fact, the regulation of International Olive Council (IOC) (2021) classified table olives according to sensory evaluation. Different types of spoilage may appear in table olives, such as putrid, zapateria, butyric, musty, rancid, or vinegary sensations (Lanza and Amoruso, 2020; Martín-Vertedor et al., 2021). Therefore, uncontrolled industrial practices could provoke the development of certain table olives alterations.

The early detection of defects during the fermentation process currently requires complex and/or time-consuming analytical techniques based on microbiological analysis, gas chromatography, or spectroscopic methods, and sensory analysis. The latter is a regularly used method to quickly detect and identify odors and spoilage microorganisms; on the contrary, the detection of volatile organic compounds is limited due to its expensive instruments, and slow, complex and voluminous analysis processes (Seesaerd et al., 2022).

Defects in food matrices are therefore determined by means of electronic devices such as the E-nose, which is used as an olfactory system through a series of sensors (Duan et al., 2021). These sensors mimic the human nose to recognize, classify, and evaluate the different volatile compounds (Siadat et al., 2014). Authors such as Sánchez et al. (2021b) used an E-nose to discriminate anomalous fermentations in Spanish-style table olives. Therefore, the objective of this work was to discriminate altered, synthetic brines inoculated with different mold strains according to their sensory attributes and volatile compound profile with the use of an E-nose.

## Materials and Methods

### Chemical Reagents

For the preparation of the synthetic brine, NaCl (Sharlau, Spain), lactic acid (Sharlau, Spain), glucose (Sharlau, Spain), and yeast extract (Condalab, Spain) were used. YPD agar (Condalab, Spain) was used for the growth of the altering molds. Commercial standard volatile organic compounds (VOC) solutions were used for the volatile organic compounds studied [propanoic acid, butanoic acid, 3,5-dimethyl-benzenemethanol, 2-methoxy-phenol, octanal, dodecanal, 2-methyl-butanoic acid, butyl ester butanoic acid, pentadecane, 3-methyl-1-butanol, 1-ethylpropyl-benzene, hexanal, heptanal, 2-nonanone, benzaldehyde, nonanal, 2,4-dimethyl-benzaldehyde, (E)-2-decenal, and a-murolene]. All these chemical reagents were purchased from Fisher Scientific (Fisher Scientific, MO, United States).

### Experiment Design

**Figure 1:**
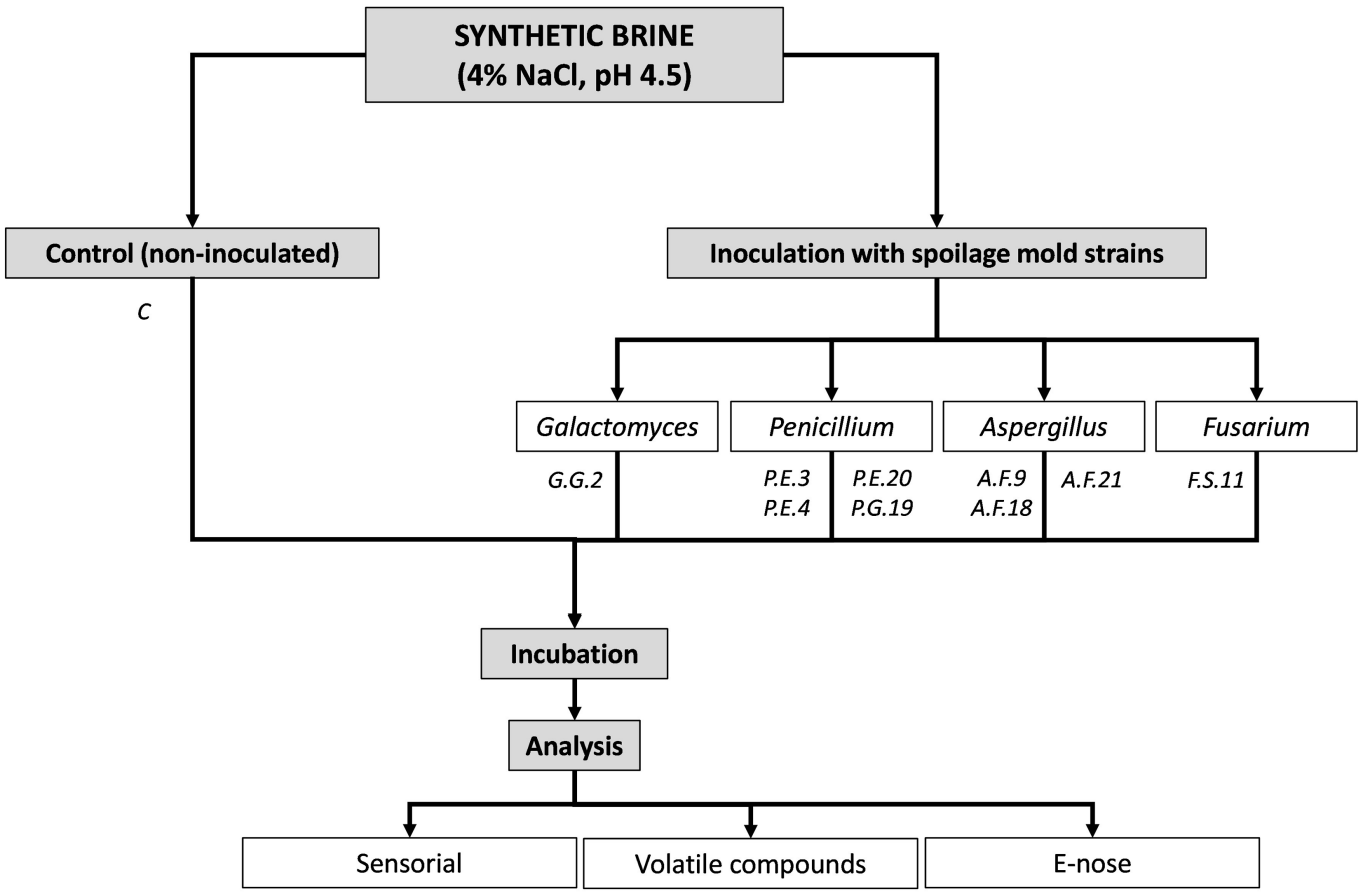
Diagram of the experimental design.

The experimental design (Figure 1) consists of eight samples for each class or species of mold strain and uninoculated synthetic brine. The total amount of samples was 80. A synthetic brine was used in this work and made up of 4% NaCl, 0.5% glucose, 0.05% yeast extract, and adjusting the pH to 4.5 with lactic acid; this brine was prepared according to Tofalo et al. (2014) with some modifications. Next, 40 ml of this brine were dispensed into 50 ml Falcon tubes and then autoclaved at 121°C for 15 min. After sterilization, the synthetic brine was inoculated with nine spoilage mold strains: one *Galactomyces geotricum* (G.G.2); three *Penicillium expansum* (P.E.3, P.E.4, and P.E.20); one *Penicillium glabrum* (P.G.19); three *Aspergillus flavus* (A.F.9, A.F.18, and A.F.21); and one *Fusarium solani* (F.S.11). These molds had been obtained and characterized by Pérez-Nevado et al. (2011) from olives during the table olive process.

Prior to the inoculation, mold strains were grown in YPD agar (Condalab, Spain) at 25°C for 10 days. The mold spores were then collected in distilled water, and the number of spores was determined using a Neubauer improved chamber; this suspension was diluted properly to inoculate a concentration of 10^5^ spores/ml in the brine. Control brines were made without inoculation. All the treatments were performed in triplicate.

The inoculated and non-inoculated synthetic brines were incubated in 50 ml Falcon tubes with a loose (air-open) cap for 15 days at 25°C in an orbital shaker at 100 rpm. Volatile compound analysis, sensory analysis and headspace measurements of the samples using an E-nose were performed at the end of the incubation. A diagram of the experiment is shown in Figure 1.

### Analyses

To carry out this study, three different types of analyses were performed on the synthetic brines inoculated with molds: sensory analysis, volatile profile, and E-nose measurements.

#### Sensory Analysis

A tasting panel was composed of eight experts from the CICYTEX research center (Extremadura, Spain) and University of Extremadura. Tasters were trained according to the IOC recommendations [International Olive Council (IOC), 2021].

For the sensory analysis of the synthetic brine, a score board was prepared. Inoculated synthetic brines were evaluated by the sensory panel on a structured scale from 0 to 10 points according to positive and negative odors perceived. The assessment of the sensory properties of the brine included aspect related to the aroma perceived to yeast, almond, sweet, mold, woody, leather, rancid, toasted, metal, and chemical. The intensity of the different odor attributes was evaluated by the trained panel, and the outcomes were expressed as average values. When the coefficient of variation was less than 20, the values were considered valid. Based on the standard International Olive Council (IOC) (2021), brines were classified according to the intensity of the defect predominantly perceived (DPP) as follows: Extra or Fancy (DPP < 3); First, 1st, Choice or Select (3 < DPP < 4.5); Second, 2nd or Standard (4.5 < DPP < 7.0); and Olives that may not be sold as table olives (DPP > 7).

#### Volatile Compound Analysis

For VOC determination, synthetic brines were filtered, and 2.0 ml were introduced into a vial with 7 ml of NaCl solution (30% p/v). The samples were introduced into polydimethylsiloxane/divinylbenzene (PDMS/DVB) StableFlex fiber (65 μm, Supelco) at 40°C for 30 min according to the methodology described by Sánchez et al. (2021a) and López-López et al. (2019). The determinations were performed using a model 456-GC triple quadrupole gas chromatograph with DB WAXETR capillary column (60 m × 0.25 mm; ID: 0.25 mm) was purchased from Agilent Technologies (Palo Alto, CA, United States). The peaks obtained were compared with the NIST reference spectral library.

#### E-Nose

The E-nose used was a small (39 mm × 33 mm), lightweight, wireless device with a low power consumption (185 mA), powered by a 3.7 V_DC_ and 2,000 mAh rechargeable LiPo battery. Its size and weight make it easy to handle, and it can be placed directly on the tasting glasses with a 3D printed case. The E-nose board has a +3.3 V_DC_ and +1.8 V_DC_ power supplies, and is governed by a PIC32MM0256GPM048 microcontroller from Microchip Technology Inc. (Chandler, AZ, United States). Among other features, this microcontroller has been chosen because of it has 256 KB of program memory, 32 KB of data memory, and several I^2^C and UART modules. The E-nose also has a Microchip’s RN4871 Bluetooth module, a UART port serial communication, and a battery charger *via* micro USB-B. The sensors used (Sánchez et al., 2021a) are BME680 from Bosch Sensortech GmbH (Germany), SGP30 from Sensirion AG (Switzerland), and CCS811 and iAQ-Core from ScioSense B.V. (The Netherlands). All of them are Metal Oxide Semiconductor (MOS) sensors, have their microprocessor, analog-to-digital converter, and I^2^C bus interface. These sensors send a total of 14 signals, distributed as follows:

- BME680: temperature (°C), pressure (hPa), humidity (% RH), and gas measurement (Ω).
- SGP30: equivalent CO_2_ (eCO_2_; ppm), total volatile organic compounds (TVOC; ppb), and the raw measurements of H_2_ and ethanol.
- CCS811: eCO_2_ (ppm), TVOC (ppb), and sensor resistance (Ω).
- iAQ-Core: eCO_2_ (ppm), TVOC (ppb), and sensor resistance (Ω).

**Figure 2:**
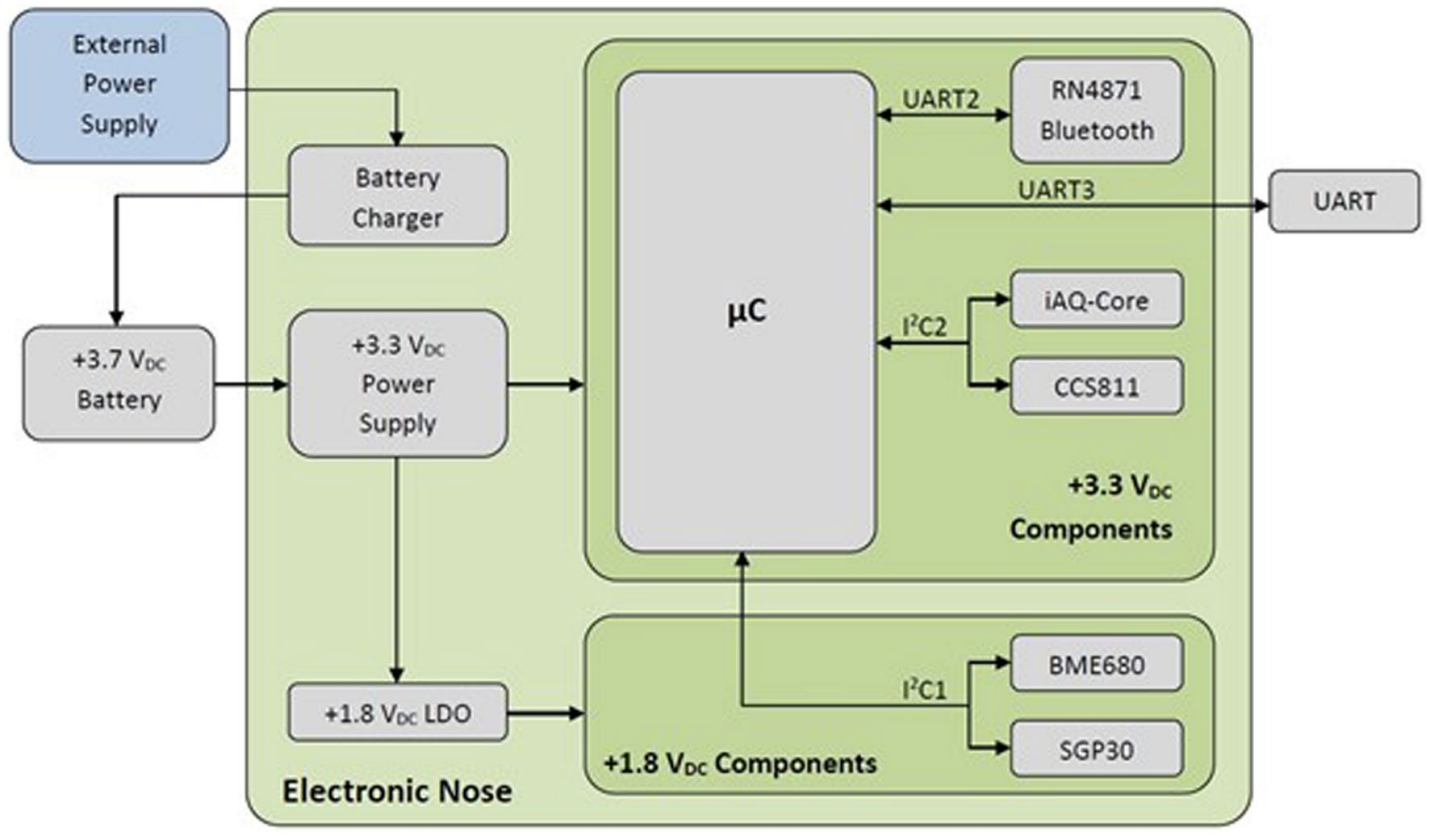
Blocks diagram of the electronic nose (E-nose).

A block diagram of the E-nose is shown in Figure 2. The data obtained are sent *via* Bluetooth to a smartphone using an ASCII based protocol, in which all signals are sent at the same time separated by tabs in a single row, and each measurement is sent every second, resulting in a table where each column corresponds to a signal and each row to a measurement. Commands to start and stop experiments have also been implemented. Finally, an Android application has been developed to get and save this data for further processing with an easy user interface.

For the aroma analysis measurements of the E-nose, the recommendations of the International Olive Council (IOC) (2021) were followed. The cups were placed in a heating block at 25°C, and 10 ml of brine were placed inside and then covered with a watch glass. The measurements were made by placing the E-nose sensors in contact with the headspace of the brine samples for 60 s and then, in the desorption phase, placing them in contact with the air contained in an empty cup for 30 s. Eight measurements were taken for each brine sample. The data obtained were further processed by the statistical tool MATLAB.

### Multivariate Data Analysis

The Principal Component Analysis (PCA) method eliminates any redundancy or correlation in the sensor responses. With the help of graphs, we could plot the principal components of the PCA of the brine samples with different molds. The values obtained from the brine samples with different molds were grouped in the graph according to their volatile compound profile.

The final objective was to automatically recognize patterns whose class we did not know. For this purpose, a supervised classification analysis called partial least squares discriminant analysis (PLS-DA) was applied (Barker and Rayens, 2003). The models were optimized with a number of latent variables equal to 7. These variables were selected through cross-validation with the leave-one-out procedure. For each model, eight samples were used for each class. A supervised classification requires prior knowledge of a group of patterns and their class, in order to subsequently obtain the model. Several PLS-DA models were built with different classification objectives. Specifically, one model was developed to discriminate between brine samples altered by strains of different molds and another to discriminate between brine samples altered by molds of the same species. The confusion matrix of the different models was constructed, and the correct predictions were calculated from the sum of the diagonal elements found in these matrices.

## Results

### Sensory Aroma of Inoculated Brines

**Figure 3:**
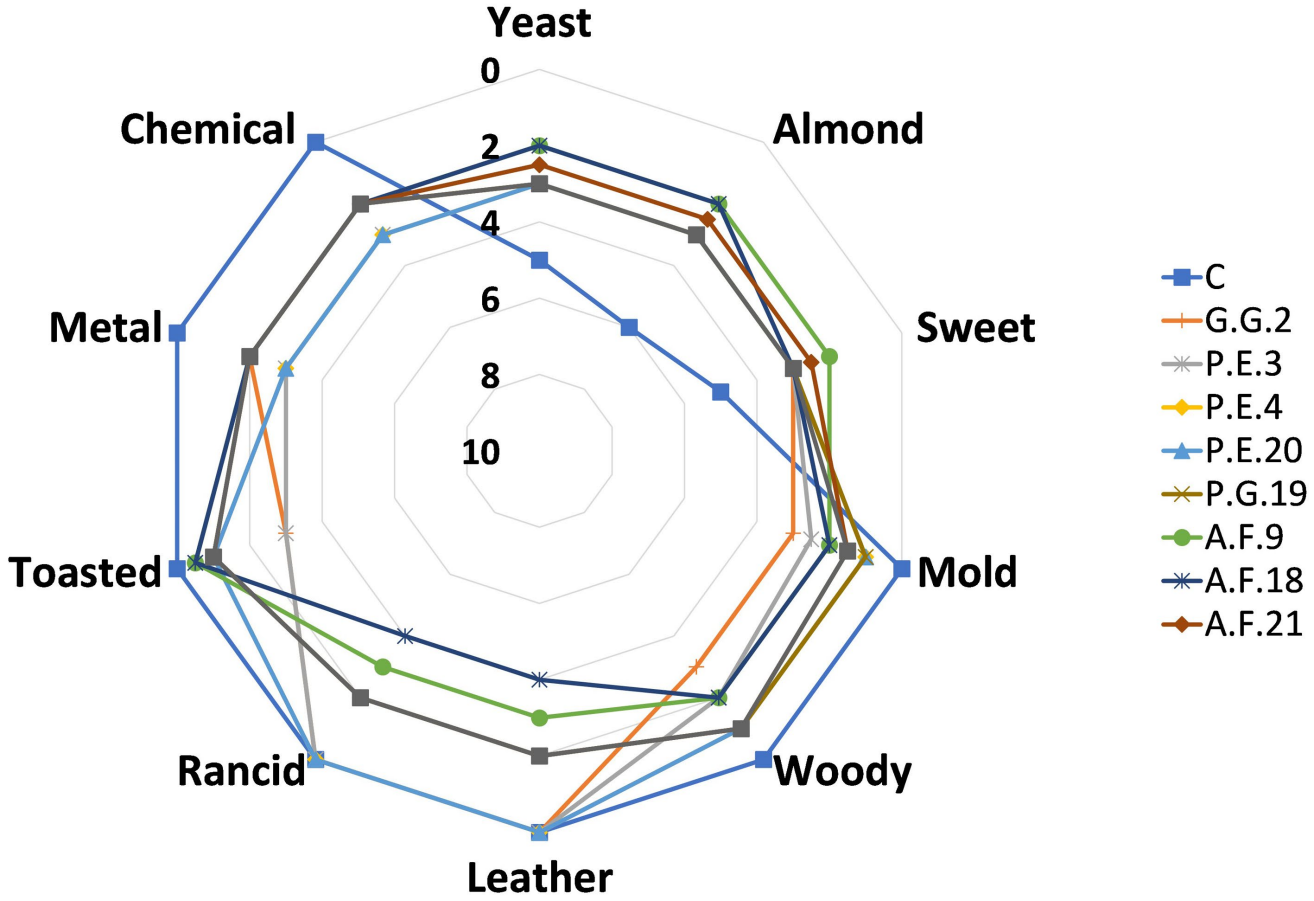
Intensity of the positive and negative attributes (mean ± SD) of synthetic brines inoculated with different mold strains.

Synthetic brines inoculated with the different mold strains were evaluated by a tasting panel that scored them according to positive and negative attributes (Figure 3). The analyzed brines were classified into different sensory categories (extra, first, and second category) according to the predominant perceived defect (PPD) intensity indicated by the International Olive Council (IOC) (2021). Different sensory profiles were obtained depending on the microorganism inoculated in the synthetic brines. The non-inoculated samples (C) did not show negative attributes but presented positive ones related to a yeast, almond, or sweet odor. The intensity of these attributes ranged from 5 to 6 points out of 10. At low concentrations, these positive attributes were also found in the inoculated ones, with the highest scores in brines with P.E.4, P.E.20, F.S.11, and P.G.19; and the lowest scores in brines with A.F.9 and A.F.18. However, the inoculated synthetic brines showed clear sensory defects related to microbial alterations. Leather and rancid attributes were the main defects found in the inoculated brines with the highest scores in A.F.9 and A.F.18 for both attributes. Other negative attributes were a metal and chemical odor, albeit in a lower quantity. The mold strains P.E.3, P.E.4, and P.E.20 showed the highest score of these attributes. Moldy, woody, and toasted odors were also detected in the inoculated synthetic brines in different concentrations. On the whole, the greatest negative sensory defects were ascribed to synthetic brines inoculated with G.G.2, P.E.3, A.F.9, and A.F.18, while the lowest values were given to P.E.4 and P.E.20.

### Volatile Compounds of Inoculated Brines

**Figure 4:**
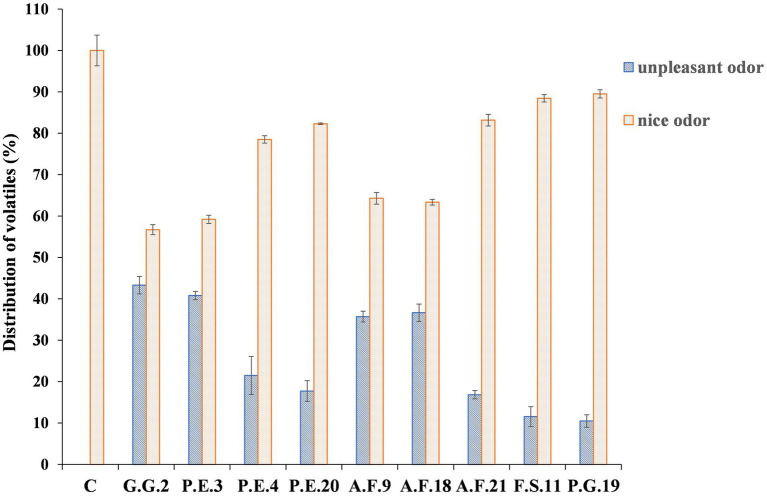
Chemical distribution of volatile compounds in synthetic brines inoculated with different mold strains.

The distribution of VOC in the control and in the inoculated brines is shown in Figure 4. A total of 19 volatile compounds were identified by gas chromatography. These compounds were classified according to their positive or negative sensory attributes. Statistically significant differences were observed between the distribution of VOCs in inoculated samples. The control (C) did not show VOCs associated with unpleasant odors. On the other hand, in inoculated brines, the microorganisms that contributed to the worst unpleasant odor intensity were G.G.2, P.E.3, A.F.9, and A.F.18, while P.E.20, A.F.21, F.S.11, and P.G.19 had the least negative odor contribution to the brines.

The VOC that contributed more to the unpleasant odor in synthetic brine inoculated with G.G.2 and P.E.3 was dodecanal, with concentrations of 23.2 and 22.4%, respectively (Table 1). In the brines inoculated with A.F.18 and A.F.9, the VOCs that contributed more to the unpleasant odor were carboxylic acids, butanoic acids (12.2 and 12.4%, respectively), and propanoic acid (10.2 and 10.5%, respectively). Other acids such as butanoic acid and propane acid were found in A.F.18 (12.3%) and in A.F.9. (12.4%). However, propane acid presented a concentration of 10.5% in A.F.18 and 10.5% in A.F.9. Another unpleasant compound synthesized during the mold’s fermentation was 2-methoxy-phenol. This was detected in higher concentrations in G.G.2 (4.9%) and in *A. flavus* strains A.F.9 and A.F.8 (3.2%). Moreover, different VOCs were detected in C brines. Nonanal (44.5%) and 2-4-dimethyl-benzaldehyde (33.4%) were the main VOCs found but other compounds such as 1-ethylpropyl-benzene (7.5%) and 2-nonanone (7.0%) were also detected. It should be noted that the percentage of these compounds decreased when the brine was inoculated by the different mold strains and in some of them, they were not detected.

**Table 1:** Content of volatile compounds (mean %, *n* = 3) obtained from brines of olives inoculated compared with olive tables (control).

	Volatile compounds	R.T (min)	C	G.G.2	P.E.3	P.E.4	P.E.20	P.G.19	A.F.9	A.F.18	A.F.21	F.S.11

*Unpleasant odor*	Propanoic acid	3.9	0.0	4.5	6.2	5.5	4.7	2.4	10.2	10.5	3.9	2.5
	Butanoic acid	8.2	0.0	5.4	7.1	6.2	4.8	3.5	12.4	12.2	5.6	4.0
	3,5-dimethyl-benzenemethanol	29.9	0.0	3.2	1.5	1.6	1.7	1.3	3.4	3.4	1.7	1.4
	2-methoxy-phenol	21.3	0.0	4.9	2.4	2.5	2.6	2.1	3.2	3.2	2.6	2.2
	Octanal	17.0	0.0	0.0	0.0	2.7	0.0	0.0	3.4	3.5	0.0	0.0
	Dodecanal	36.4	0.0	23.2	22.4	1.7	3.1	0.3	0.1	0.3	0.4	0.4
	2-methyl-butanoic acid	10.1	0.0	0.0	0.0	0.0	0.0	0.0	2.5	2.7	1.8	0.0
	butyl ester butanoic acid	34.8	0.0	2.1	0.8	1.1	0.4	0.4	0.2	0.7	0.7	0.5
	Pentadecane	36.0	0.0	0.0	0.3	0.3	0.4	0.4	0.3	0.3	0.3	0.6
*Positive odor*	3-methyl-1-Butanol	4.4	0.0	3.1	4.3	3.8	3.2	3.7	10.3	10.0	3.6	3.7
	1-ethylpropyl-benzene	30.6	7.5	2.7	1.1	1.2	0.0	0.0	0.0	0.0	0.0	0.0
	Hexanal	6.7	1.0	0.0	0.0	1.0	0.2	0.2	0.4	0.9	1.0	1.1
	Heptanal	11.6	1.1	0.0	0.0	0.2	0.9	0.6	0.1	0.3	0.0	0.4
	2-Nonanone	13.1	7.0	5.0	5.8	3.4	6.9	6.1	3.5	3.3	3.1	7.2
	Benzaldehyde	14.8	5.5	0.0	0.0	0.0	0.8	3.4	0.0	0.7	0.9	3.2
	Nonanal	22.3	44.5	20.3	20.8	42.3	44.6	38.7	23.5	23.6	44.1	44.4
	2,4-dimethyl-benzaldehyde	27.8	33.4	20.6	21.3	22.2	21.5	34.2	22.9	20.5	27.0	25.6
	(E)-2-Decenal	30.0	0.0	3.0	4.1	3.8	3.2	1.6	3.5	3.0	2.7	1.7
	α-muurolene	40.0	0.0	2.0	1.8	0.7	1.1	1.0	0.1	1.0	1.0	1.0

RT, retention time.

### Discrimination of Inoculated Synthetic Brines With E-Nose

**Figure 5:**
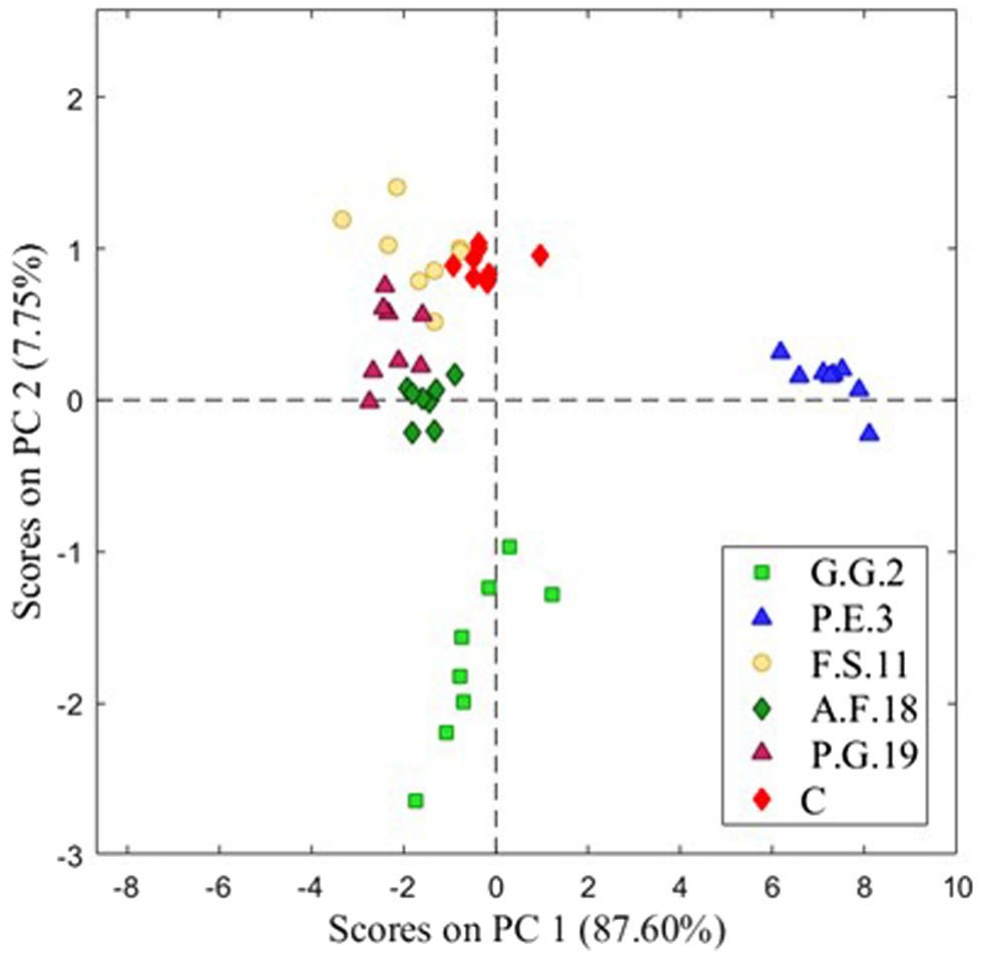
Score plot of the Principal Component Analysis (PCA) analysis for control inoculated by different mold strains.

Brine samples inoculated with different mold strains were analyzed with an E-nose and data obtained were analyzed using a PCA (Figure 5). A separation between C and inoculated brines could be observed. The PCA results showed that 87.60% of the total variance of the data was explained by PC1 and 7.75% by PC2. The model based on the first two components showed a clear differentiation of C and the inoculated brine samples.

After the application of a PLS-DA classification analysis and leave-one-out cross-validation of the data, we obtained the confusion matrix shown in Table 2. The sum of the diagonal elements of the confusion matrix gave a classification success rate of 93.5%. These results prove the ability and accuracy of the E-nose to discriminate between different brines with alterations caused by different mold strains.

**Table 2:** Confusion matrix obtained from eight samples of each class through partial least squares discriminant analysis (PLS-DA) for discrimination between different synthetic brine inoculated by molds.

Predicted class (%)						
Real class	C	G.C.2	P.E.3	F.S.11	A.F.18	P.G.19
C	16.6	0	0	0	0	0
G.C.2	0	16.6	0	0	0	0
P.E.3	0	0	16.6	0	0	0
F.S.11	0	0	0	16.6	0	0
A.F.18	0	0	0	0	12.5	2.0
P.G.19	0	0	0	0	4.2	14.6

**Figure 6:**
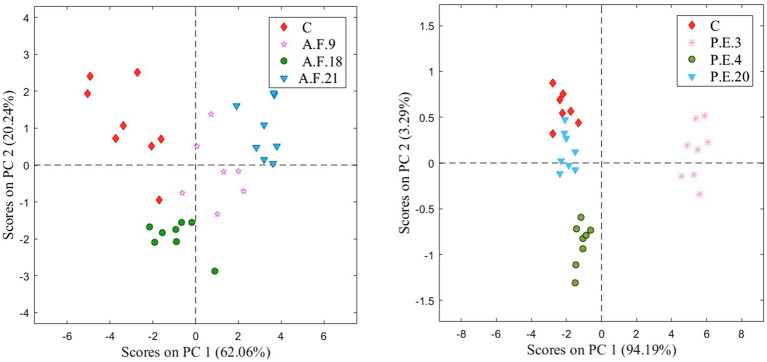
Score plot of the PCA for brine inoculated by the *A. flavus* species (left), and the score plot of the PCA for brine inoculated by *P. expansum* species (right).

In addition, the response of the E-nose to brine samples non-inoculated and inoculated with several mold strains of *A. flavus* and *P. expansum* was analyzed using a PCA (Figure 6). In this figure, it is possible to differentiate non-inoculated brines from inoculated ones. The first and second principal components (PC1 and PC2) were sufficient to visualize the clustering of data and explained 82.2 and 97.4% of the total variance of *A. flavus* and *P. expansum*, respectively. Subsequently, the results obtained after applying the PLS-DA (Table 3) showed a 78% hit rate for *A. flavus* strains and a 93% hit rate for *P. expansum*.

**Table 3:** Confusion matrix obtained from eight samples of each class through PLS-DA for discrimination between synthetic brine inoculated by *Aspergillus flavus* and *Penicillium expansum* strains.

Predicted class (%)									
*A. flavus*					*P. expansum*				
Real class	C	A.F.9	A.F.18	A.F.21	Real class	C	P.E.3	P.E.4	P.E.8
C	18.7	3.1	3.1	0	C	21.9	0	0	3.1
A.F.9	3.1	15.6	3.1	0	P.E.3	0	25.0	0	0
A.F.18	3.1	3.1	18.7	0	P.E.4	0	0	25.0	0
A.F.21	0	3.1	0	25.0	P.E.8	3.1	0	0	21.9

## Discussion

According to the PPD established by the International Olive Council [International Olive Council (IOC), 2021], the metabolism of mold strains could have caused sensory defects in the synthetic brines. In contrast, when evaluated by the tasting panel, the non-inoculated synthetic brines presented positive sensory attributes that could be due to the yeast extract added to the brine to improve the microbial growth. It should be noted that these positive attributes were also present in inoculated brines but in all cases to a lesser extent. The decrease in their concentration in brines with molds may be due to the microbial metabolism caused during their development.

Tasters detected many negative attributes in different concentrations in the inoculated brines. In this sense, most of the molds (G.G.2, P.E.3, P.E.4, P.E.20, A.F.9, and A.F.18) provoked some alteration in the brine and were classified into the first category as the DPP had a score of 4 (3 < DPP ≤ 4.5). The rest of the mold strains (A.F.21, F.S.11, and P.G.19) were the only mold strains classified into the extra category (PPD ≤ 3) although they presented sensory defects at low concentrations. Furthermore, the PPD in the synthetic brines inoculated was less than 6, thus it could be marketed.

In the Spanish-style table olive process, the sensorial properties can be modified by the formation of volatile and non-volatile aromatic compounds (Mas et al., 2014). These include peptides, amino acids, vitamins, minerals, and polyphenols, all of which can contribute to the sensory aroma. The non-inoculated brines (C) presented VOCs responsible for positive aromas. This is the case of 3-methyl-1-butanol, a product formed in alcoholic fermentations by microorganisms (Pietruska et al., 2010), and with a fruity aroma such as apples and bananas (Shen et al., 2022). The carbonyl compounds found in the non-inoculated samples are thought to be related to the commercial yeast extract added during the preparation of the brines. The prominent compound was Nonanal whose concentration varied depending on the microorganism inoculated in the brine; P.E.4, P.E.20, A.F.21, and F.S.11 presented similar concentrations to C; in the rest of the mold strains, the concentration of nonanal was reduced by half compared to C. This compound, like other aromatic aldehydes found in essential oils of various plants, has antifungal properties such as the inhibition of *A. flavus* (Li et al., 2021). A further compound that provides positive attributes is 2,4-dimethyl-benzaldehyde, related to aroma of cherry, almond, and vanilla (Zhang et al., 2021). Overall, the concentrations in inoculated brines were lower compared to the control, except for P.G.19, which increased. In the case of aromatic ketones such as 2-nonanone, only its concentration in F.S.11 increased in the brines in relation to C. This aromatic carbonyl compound also exhibits antimicrobial properties (Mahmound et al., 2020).

On the other hand, VOCs associated with unpleasant aromas were found in the brines inoculated by different mold strains. The microorganisms produced two short-chain carboxylic acids, namely propanoic acid and butanoic acid. The first has a cheesy odor and the second produces a buttery and cheesy odor (Liu et al., 2022). A.F.9 and A.F.18 were the main contributors to the formation of these carboxylic acids. In general, molds generate undesirable products during fermentation processes (Sánchez et al., 2021b), and at the same time cause serious problems to food safety and human health (Gong et al., 2019). Two derivatives of butanoic acid found in our study were 2-methyl-butanoic acid and butyl ester-butanoic acid. These are associated with unpleasant odors in brines; the first derivative has a cheesy, sweaty, and sharp odor (Tachihara et al., 2006). The butyl ester-butanoic acid is a compound synthesized by microorganisms through enzymes acyl transferases and lipases that play an important role in butanol esterification (Noh et al., 2019). In our study, these compounds were synthesized by the strain of *G. galactomyces* G.G.2. In other studies (Eliskases-Lechner et al., 2011), *Geotrichum* was heavily involved in the flavor and aroma of cheese, and was responsible for the deterioration and reduction of the shelf-life of fermented products derived from milk. The dodecanal aldehyde compound was found in different concentrations in all the inoculated brines. G.G.2 and P.E.3 were the microorganisms capable of synthesizing the highest amounts of this compound. Microorganisms could be used to synthesize aldehydes as an alternative to traditional synthetic routes (Kunjapur and Prather, 2015).

In this way, the different mold strains used to inoculate synthetic brines were discriminated with the E-nose. This discrimination could be due to the different aroma profiles present in the inoculated brine. This electronic tool was even able to discriminate brine samples inoculated with several strains of *A. flavus* and *P. expansum*. Few references exist on the discrimination of olives altered by mold using an E-nose. Sánchez et al. (2021b) discriminated table olive defects produced by different microorganisms, such as zapateria, butyric, putrid, and mold during the fermentation period of Spanish-style table olives. There are studies that demonstrated the usefulness of sensory-based E-nose systems to the fungal detection and identification of associated species (Mota et al., 2021); and to develop a detection system to differentiate fungus using an E-nose device (Loulier et al., 2020). Therefore, the results obtained in this study prove that an E-nose, combined with chemometric analysis, is a powerful tool with an analytical capacity to discriminate VOCs produced by different mold strains. Moreover, it is a fast, precise and low-cost method compared to classic analysis techniques since it is non-destructive and does not require highly qualified personnel (Nishi et al., 2015).

## Conclusion

The inoculated mold strains produced negative sensory defects in the synthetic brines. The main defects detected were mold, rancid, and leather, most of which were classified into the first category established by the IOC. The inoculated brines showed a different profile of volatile compounds associated with unpleasant aromas that depended on the inoculated mold strain; dodecanal was the VOC with the highest content, followed by butanoic acid and propanoic acid. Therefore, taking into account the sensory analysis and VOC, the mold strains that caused the most unpleasant effects in the synthetic brines were G.G.2, P.E.3, A.F.9, and A.F.18. These defects were also discriminated by using an E-nose, a powerful tool capable of differentiating the aroma of the synthetic brines inoculated with different mold strains. Combined with other chemometric tools, this easy-to-use, low-cost device can discriminate samples according to incipient alterations caused by molds, thereby confirming its suitability to detect different alterations during the fermentation process in Spanish-style table olives at industrial level, although further studies would be required to prove this.

## Data Availability Statement

The raw data supporting the conclusions of this article will be made available by the authors, without undue reservation.

## Author Contributions

RS, FP-N, IM-F, and DM-V contributed to the conceptualization of the study. RS, JL, and FM contributed to data curation and methodology. Formal analysis was performed by RS, IM-F, and DM-V. DM-V and FP-N acquired the funding. DM-V carried out the project administration. RS, FP-N, and DM-V performed the investigation and organized the resources. JL and DM-V carried out the supervision. JL, RS, FM, and DM-V contributed to the validation. RS and DM-V contributed to the visualization. RS, IM-F, FM, JL, and DM-V wrote the original draft. RS, IM-F, JL, and FP-N contributed to the writing—review and editing of the final manuscript. All authors contributed to the article and approved the submitted version.

## Funding

This work was supported by the Junta de Extremadura (Spain) and the European Regional Development Fund (grant number: GR21121).

## Conflict of Interest

The authors declare that the research was conducted in the absence of any commercial or financial relationships that could be construed as a potential conflict of interest.

## Publisher’s Note

All claims expressed in this article are solely those of the authors and do not necessarily represent those of their affiliated organizations, or those of the publisher, the editors and the reviewers. Any product that may be evaluated in this article, or claim that may be made by its manufacturer, is not guaranteed or endorsed by the publisher.
